# Supplementary material for: Predicting visual function by interpreting a neuronal wiring diagram
Source: Nature. 2024 Oct 2;634(8032):113–23. doi: 10.1038/s41586-024-07953-5 (PMC11446822; doi:10.1038/s41586-024-07953-5)
Supplement: Supplementary file 6 — CRF and ERF predictions for individual TmY4 and TmY9 cells. Analogous to Supplementary Data 3, but for TmY target types. Shown are the top four monosynaptic pathways, the strongest pathway passing through each of the top ten intermediary types (ranking from Extended Data Fig. 7), and the trisynaptic pathway Tm1–TmY–Dm3–TmY (see the section entitled Prediction of spatial normalization). [file 41586_2024_7953_MOESM6_ESM.zip › DataS4/TmY9q/720575940627523371.pdf]

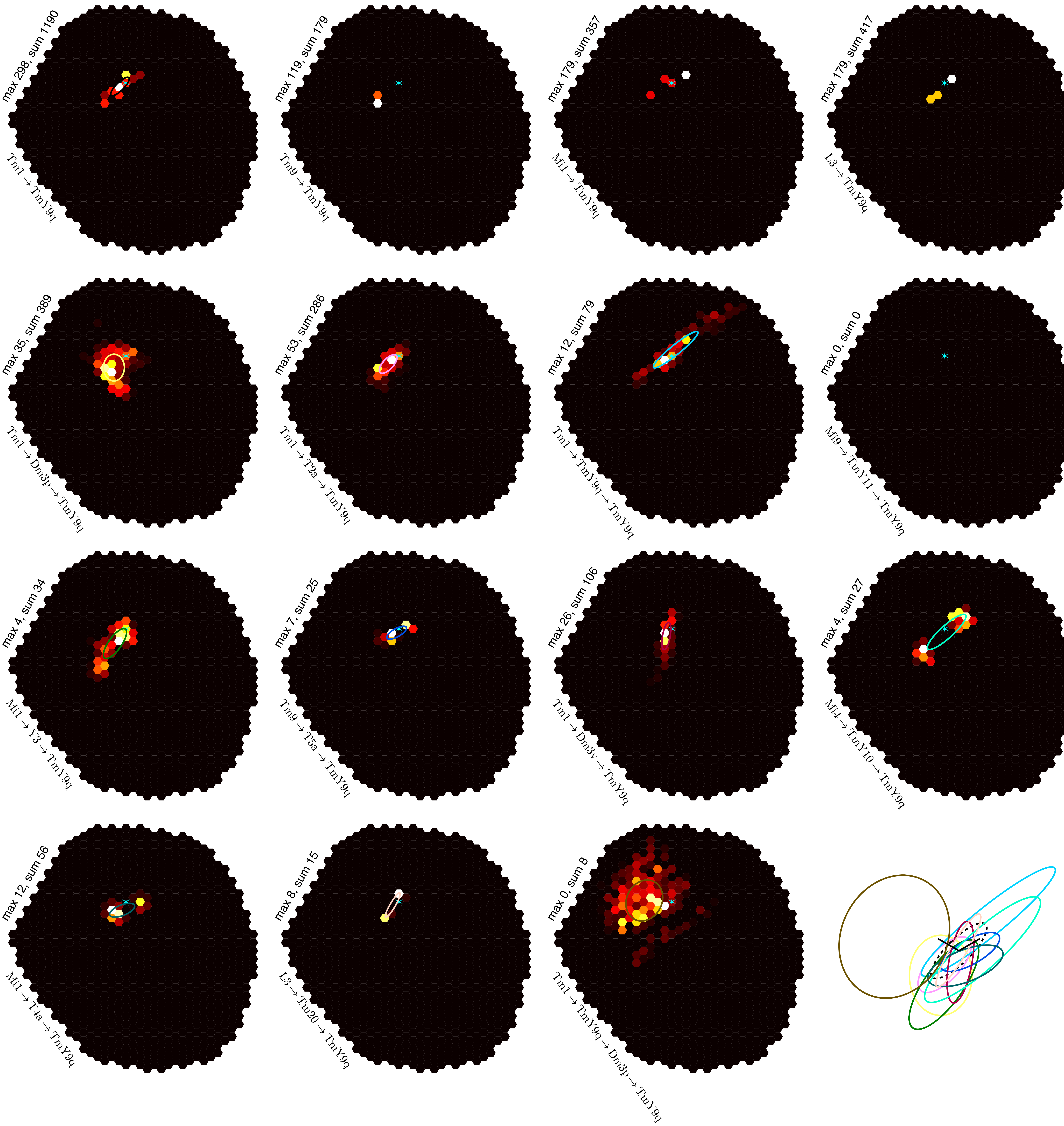
max 298, sum 1190
Tm1 → TmY9q
max 119, sum 179
Tm9 → TmY9q
max 179, sum 357
Mi1 → TmY9q
max 179, sum 417
L3 → TmY9q
max 35, sum 389
Tm1 → Dm3p → TmY9q
max 53, sum 286
Tm1 → T2a → TmY9q
max 12, sum 79
Tm1 → TmY9q → TmY9q
max 0, sum 0
Mi9 → TmY11 → TmY9q
max 4, sum 34
Mi1 → Y3 → TmY9q
max 7, sum 25
Tm9 → T5a → TmY9q
max 26, sum 106
Tm1 → Dm3v → TmY9q
max 4, sum 27
Mi4 → TmY10 → TmY9q
max 12, sum 56
Mi1 → T4a → TmY9q
max 8, sum 15
L3 → Tm20 → TmY9q
max 0, sum 8
Tm1 → TmY9q → Dm3p → TmY9q
